# Combined endoscopic and laparoscopic management of mesh erosion following vertical banded gastroplasty: a case report and literature review

**DOI:** 10.3389/fmed.2026.1728448

**Published:** 2026-02-06

**Authors:** Jho-Jhen Li, Tsung-Hsien Chen, Chu-Kuang Chou, Sheng-Shih Chen

**Affiliations:** 1 Department of General Surgery, Kaohsiung Veterans General Hospital, Kaohsiung, Taiwan; 2 Department of Internal Medicine, Ditmanson Medical Foundation Chia-Yi Christian Hospital, Chiayi, Taiwan; 3 Division of Gastroenterology and Hepatology, Department of Internal Medicine, Ditmanson Medical Foundation Chia-Yi Christian Hospital, Chiayi, Taiwan; 4 Obesity Center, Ditmanson Medical Foundation Chia-Yi Christian Hospital, Chiayi, Taiwan; 5 Metabolic and Bariatric Center and Division of Surgical Critical Care, Kaohsiung Veterans General Hospital, Kaohsiung, Taiwan

**Keywords:** endoscopic removal, mesh erosion, revisional bariatric surgery, single anastomosis sleeve-ileal bypass, vertical banded gastroplasty

## Abstract

Vertical banded gastroplasty (VBG) has largely been abandoned due to late complications, including the rare but serious erosion of prosthetic mesh into the gastric lumen. We report the case of a 46-year-old woman who developed progressive epigastric pain, vomiting, and weight regain two decades after VBG. Endoscopic and radiologic evaluations revealed intragastric erosion of a polypropylene mesh, which was successfully removed endoscopically without complication. Persistent symptoms and inadequate weight control, attributed to altered gastric anatomy, necessitated staged revisional surgery. The patient subsequently underwent laparoscopic sleeve gastrectomy followed by single-anastomosis sleeve–ileal bypass, achieving sustained symptom resolution and satisfactory weight control over an 18-month follow-up period. This case supports a combined endoscopic and surgical approach for managing complex sequelae of VBG.

## 1 Introduction

**FIGURE 1:**
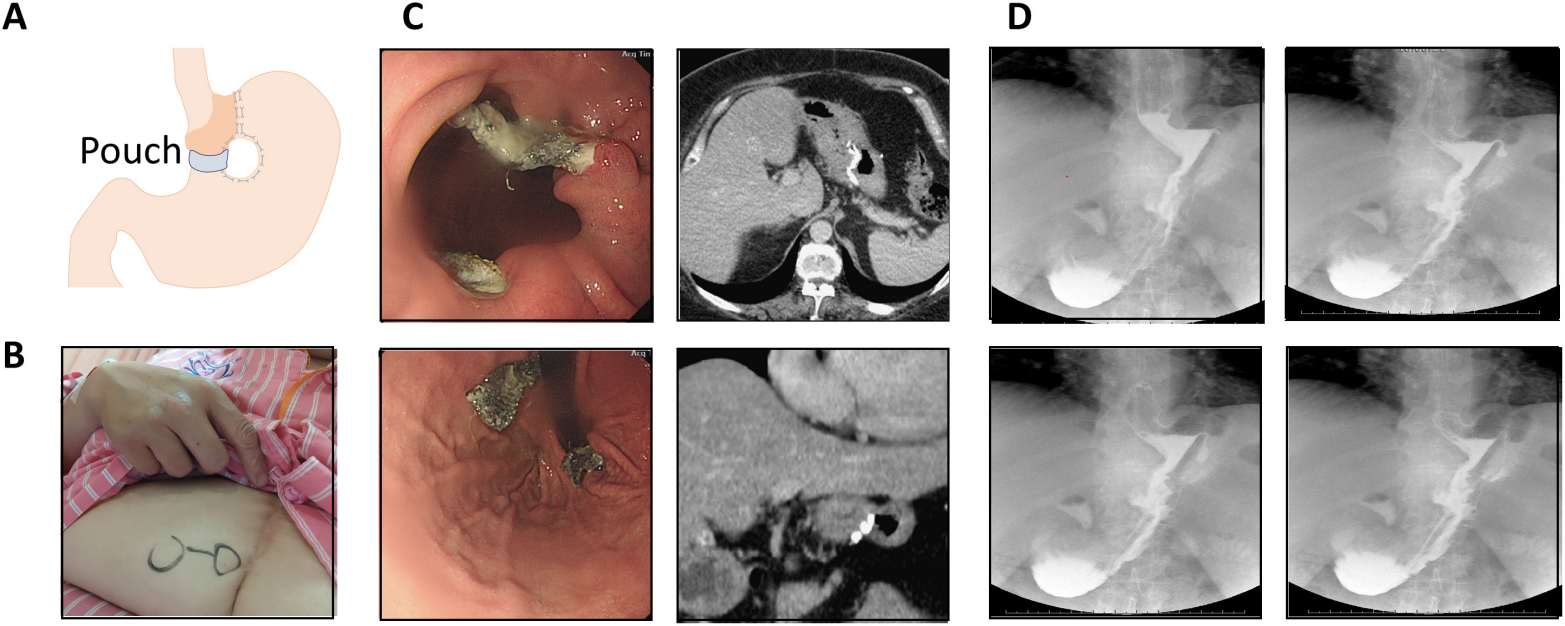
Preoperative evaluation. **(A)** Schematic illustration of vertical banded gastroplasty (VBG). **(B)** Photograph showing a vertical midline laparotomy scar from prior surgery. **(C)** Retroflexed endoscopic view revealing polypropylene mesh erosion into the gastric lumen, approximately 7 cm distal to the gastroesophageal junction (GEJ). Corresponding computed tomography (CT) confirmed complete intragastric mesh erosion. **(D)** Upper gastrointestinal contrast series showing contrast leakage into the stapled gastric pouch, suggestive of a fistulous tract.

Vertical banded gastroplasty (VBG) (Figure 1A), first introduced by Mason in 1982, was once a widely used restrictive bariatric procedure designed to promote weight loss through gastric volume reduction and outlet restriction (1). The technique involves vertical stapling to create a small proximal gastric pouch, combined with placement of a non-adjustable prosthetic band at the pouch outlet to limit food passage and induce early satiety (2). Although initially effective, long-term follow-up studies demonstrated suboptimal durability and a high incidence of late complications, leading to the decline and eventual abandonment of the procedure (3, 4). Commonly reported adverse outcomes include gastroesophageal reflux disease (GERD), staple-line disruption, gastric outlet stenosis, chronic nausea and vomiting, and significant weight regain (3, 5).

A less frequent but clinically significant late complication of VBG is erosion of the prosthetic mesh or band into the gastric lumen, which typically presents many years after the index operation. Mesh erosion poses diagnostic and therapeutic challenges due to its insidious and non-specific clinical presentation, including epigastric pain, emesis, gastrointestinal bleeding, and recurrent weight gain (6, 7).

Endoscopy remains the diagnostic modality of choice, with contrast radiography serving a complementary role. Historically, management required surgical removal; however, advances in therapeutic endoscopy have enabled endoscopic extraction to emerge as a minimally invasive alternative in selected patients (8). Nonetheless, reported experience remains limited, particularly in Asian populations, and there is no established consensus regarding optimal management, especially in cases complicated by anatomical disruption or weight recurrence.

In this report, we present a case from Taiwan of delayed polypropylene mesh erosion occurring two decades after VBG. The patient was successfully treated using a staged approach that included endoscopic mesh removal followed by laparoscopic revisional bariatric surgery. This case demonstrates the feasibility of a combined endoscopic and surgical strategy for managing complex late sequelae of obsolete bariatric procedures and highlights the importance of individualized, multidisciplinary decision-making.

## 2 Case presentation

A 46-year-old woman with a history of morbid obesity and type 2 diabetes mellitus (T2DM) underwent VBG 20 years earlier. As shown in Figure 1B, a vertical midline laparotomy scar was present. She initially achieved substantial weight loss; however, 6 years prior to presentation, she developed progressive epigastric pain, nausea, and vomiting. These symptoms worsened over the subsequent 2 years and were associated with significant weight regain. Upper gastrointestinal endoscopy performed at an outside facility revealed a foreign body within the gastric lumen and Los Angeles grade C reflux esophagitis. She was referred to our institution for further evaluation and management.

Repeat upper endoscopy confirmed a foreign body eroding into the gastric cavity. Contrast-enhanced abdominal computed tomography demonstrated intragastric erosion of a polypropylene mesh (Figure 1C). Additionally, an upper gastrointestinal contrast study revealed a communication between the gastric pouch and the excluded remnant stomach, consistent with a small gastro-gastric fistula (Figure 1D), which was considered a potential contributor to her weight regain.

**FIGURE 2:**
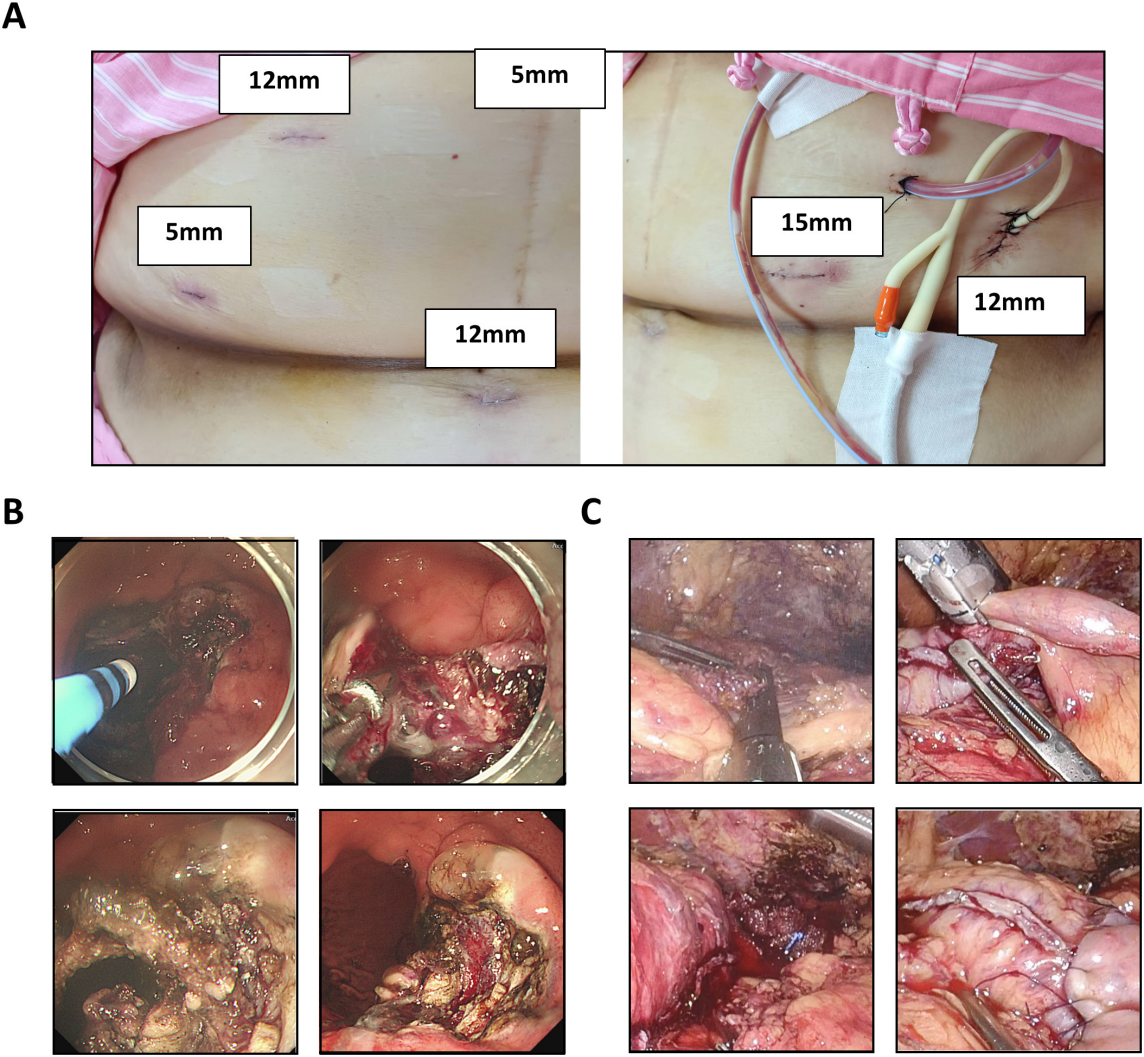
Intraoperative and Follow-up Findings. **(A)** Port placement configuration consisting of two 5-mm ports, one 15-mm port, and three 12-mm ports. **(B)** Endoscopic management of mesh erosion. Argon plasma coagulation and diathermy were used to dissect the mucosal and muscular layers for safe mesh removal. **(C)** Intraoperative image following completion of laparoscopic single anastomosis sleeve-ileal (SASI) bypass.

A combined endoscopic and laparoscopic intervention was undertaken. Under general anesthesia in the left lateral decubitus position, endoscopic evaluation identified mesh erosion through the gastric mucosa. Port placement consisted of two 5-mm ports, one 15-mm port, and three 12-mm ports (Figure 2A). Argon plasma coagulation and diathermy were used to dissect and mobilize the eroded polypropylene mesh, which was removed endoscopically without evidence of perforation (Figure 2B).

The patient was subsequently repositioned supine for laparoscopic surgery. Pneumoperitoneum was achieved using a five-trocar technique. Laparoscopic adhesiolysis was performed, followed by sleeve gastrectomy (SG) using a linear stapler (Endo GIA). Given the patient’s weight regain and concurrent gastroesophageal reflux, a single-anastomosis sleeve-ileal (SASI) bypass was constructed, with the ileal limb anastomosed 150 cm distal to the ligament of Treitz. A feeding jejunostomy and prophylactic cholecystectomy were also performed. A closed-suction drain was placed along the staple line (Figure 2C). The SG and SASI bypass were completed during the same operative session as a planned single-stage revisional procedure.

**FIGURE 3:**
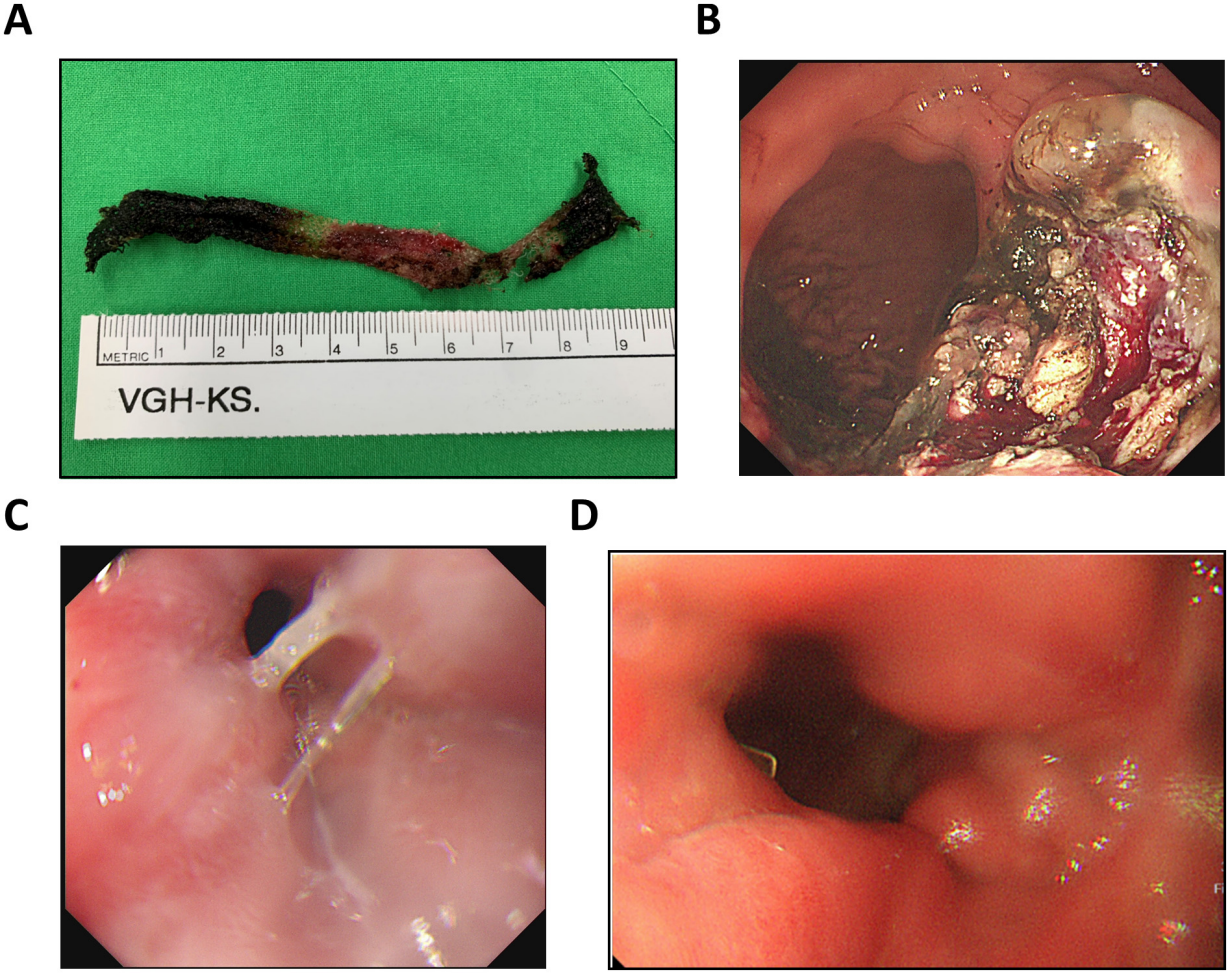
Intraoperative and endoscopic assessment. **(A)** The retrieved 10-cm polypropylene mesh following complete endoscopic removal. **(B)** Endoscopic appearance after mesh removal and prior to creation of the gastric sleeve. **(C)** Endoscopic view showing a non-traversable gastro-gastric fistula. **(D)** Follow-up endoscopy at three months postoperatively showing healed gastric mucosa at the previous site of mesh erosion, with resolution of ulceration.

The retrieved 10-cm polypropylene mesh following complete endoscopic removal is shown in Figure 3A. Endoscopic evaluation following mesh removal and prior to creation of the gastric sleeve demonstrated the operative field (Figure 3B). A non-traversable gastro-gastric fistula was identified on endoscopy (Figure 3C). Estimated intraoperative blood loss was 100 mL, and total anesthesia time was 436 min. Postoperatively, the patient did not require admission to the intensive care unit.

Enteral feeding via jejunostomy commenced on postoperative day (POD) 2. Following contrast evaluation confirming intact anastomoses and absence of leakage, clear liquids were initiated orally on POD 7. Both the surgical drain and feeding jejunostomy tube were removed on POD 9, and the patient was discharged in stable condition on POD 10, tolerating oral intake without complications.

The patient was followed for 18 months postoperatively. Her body weight decreased from 129.5 to 93 kg, demonstrating sustained weight reduction. Glycemic control improved markedly, with hemoglobin A1c decreasing from 9.6% preoperatively to 6.3%. Serum lipid parameters also normalized (total cholesterol: 205–177 mg/dL; triglycerides: 187–117 mg/dL). Surveillance upper endoscopy at 3 months (Figure 3D) demonstrated complete mucosal healing at the prior site of mesh erosion and significant resolution of the previously observed gastric ulcer. Overall, the combined endoscopic and laparoscopic approach effectively addressed mesh erosion and gastro-gastric fistula, alleviated chronic gastrointestinal symptoms, and improved metabolic indices.

## 3 Discussion

Mesh erosion following VBG is a rare but clinically significant long-term complication, with a reported incidence of 0.5%–3% (4, 9, 10). It may present many years after the index surgery, reflecting its chronic and progressive nature. The pathogenesis is multifactorial, involving chronic mechanical pressure, gastric wall ischemia, biofilm-related infection, persistent foreign body inflammation, and technical factors such as improper mesh tension or staple-line disruption (4, 9, 11, 12). Clinical manifestations are often insidious and non-specific, including epigastric pain, nausea, vomiting, dysphagia, hematemesis, halitosis, and recurrent GERD, frequently leading to delayed diagnosis (13–15).

Endoscopic removal of eroded VBG mesh is increasingly supported as a first-line treatment because of its minimally invasive nature, low perioperative morbidity, and high technical success rate (9, 16). Techniques such as argon plasma coagulation, diathermy, endoscopic scissors, and snares have been reported for mesh fragmentation and retrieval (9, 14, 16). Endoscopic intervention avoids the risks associated with laparotomy in reoperative bariatric surgery (9, 17); however, it does not address associated anatomical abnormalities such as pouch dilation, staple-line dehiscence, or gastro-gastric fistula, which are common contributors to weight regain (13, 14, 18). Consequently, endoscopic management is often palliative when structural defects coexist.

Revisional metabolic and bariatric surgery is indicated in patients with mechanical complications, uncontrolled GERD, or significant weight regain after VBG. Among available options, SASI bypass has demonstrated durable weight-loss and metabolic benefits, including favorable outcomes in T2DM remission, with acceptable perioperative morbidity (19–23). SASI provides a balance of restrictive and malabsorptive mechanisms, making it a technically feasible option in the revisional setting (24). SG may be considered when focal resection is required; however, SG alone may aggravate reflux symptoms in selected patients. Although conversion from VBG is technically challenging favorable outcomes have been reported in experienced centers (13, 19, 20).

**FIGURE 4:**
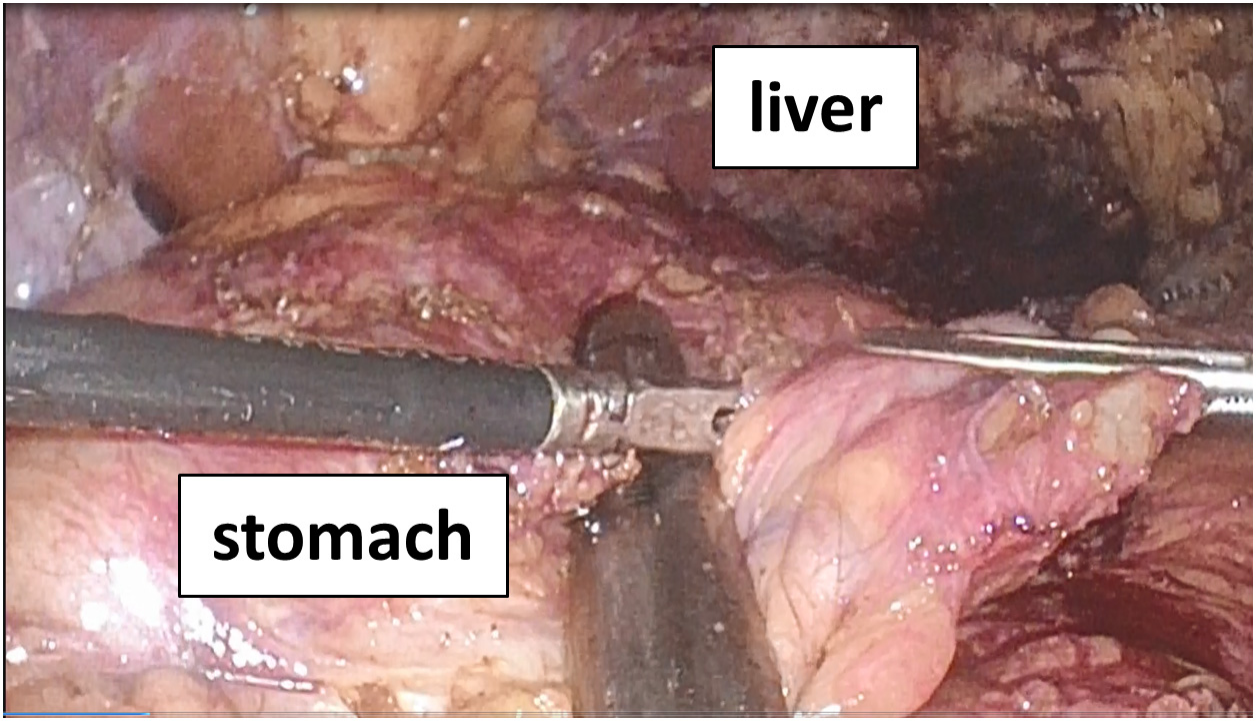
Intraoperative findings. Dense adhesions were observed along the lesser curvature, with particularly severe fibrotic adhesions attributed to prior placement of the vertical banded gastroplasty (VBG) mesh.

From a surgical perspective, VBG utilizing polypropylene mesh is associated with dense adhesions along the lesser curvature, often in close proximity to the left gastric artery. Consequently, open surgical mesh removal carries a substantial risk of catastrophic hemorrhage. Furthermore, endoscopic evaluation demonstrated that approximately two-thirds of the mesh had migrated into the gastric lumen. In this context, a laparoscopic approach may be limited, as the mesh may not be readily identifiable from the peritoneal side. In addition, the patient’s history of prior open surgery had resulted in extensive intra-abdominal adhesions, particularly along the lesser curvature attributable to the VBG mesh (Figure 4).

In the present case, a staged approach was adopted, consisting of endoscopic mesh removal followed by laparoscopic SG with conversion to SASI. This strategy was guided by both anatomical and metabolic indications: SG facilitated resection of the gastro-gastric fistula and gastric remodeling, while SASI addressed persistent GERD and provided metabolic benefit. Revisional bariatric surgery carries higher complication rates due to prior scarring and altered anatomy (14, 17); nevertheless, a staged hybrid approach may reduce operative risk by improving visualization and minimizing gastric wall tension. In our case, this strategy resulted in safe mesh retrieval and effective reconstruction without major postoperative complications.

To our knowledge, this is the first reported case in Taiwan of hybrid endoscopic and laparoscopic management of VBG mesh erosion. Recent reviews and expert consensus recommend individualized, multidisciplinary management, supporting endoscopic extraction as the initial strategy, with surgical revision reserved for anatomical abnormalities or failed endoscopic treatment (25).

## 4 Conclusion

Endoscopic removal of eroded meshis a safe, effective, and minimally invasive strategy for managing mesh erosion following vertical banded gastroplasty. However, in patients with concomitant complications—such as GERD, fistula formation, or significant weight regain—endoscopic therapy alone may be insufficient. In these cases, a combined approach incorporating revisional bariatric procedures, including SG or SASI, can provide more comprehensive symptom resolution, metabolic improvement, and sustained weight loss. Careful multidisciplinary assessment and individualized treatment planning are essential to optimize outcomes in this complex patient population.

## Data Availability

The original contributions presented in this study are included in this article/supplementary material, further inquiries can be directed to the corresponding authors.
